# Retinal venular tortuosity and fractal dimension predict incident retinopathy in adults with type 2 diabetes: the Edinburgh Type 2 Diabetes Study

**DOI:** 10.1007/s00125-021-05388-5

**Published:** 2021-01-30

**Authors:** Rachel B. Forster, Emmanuel Sandoval Garcia, Anniek J. Sluiman, Sheila M. Grecian, Stela McLachlan, Tom J. MacGillivray, Mark W. J. Strachan, Jackie F. Price, on behalf of the Edinburgh Type 2 Diabetes Study (ET2DS) investigators

**Affiliations:** 1 Usher Institute, University of Edinburgh, Edinburgh, UK; 2 Centre for Clinical Brain Sciences, University of Edinburgh, Edinburgh, UK; 3 Metabolic Unit, Western General Hospital, Edinburgh, UK

**Keywords:** Diabetic retinopathy, Digital retinal imaging, Epidemiology, Logistic regression, Microvascular disease, Prediction modelling, Prospective cohort, Retinal vessel traits, Type 2 diabetes, Vascular complications

## Abstract

**Aims/hypothesis:**

Our aim was to determine whether a range of prespecified retinal vessel traits were associated with incident diabetic retinopathy in adults with type 2 diabetes.

**Methods:**

In the prospective observational cohort Edinburgh Type 2 Diabetes Study of 1066 adults with type 2 diabetes, aged 60–75 years at recruitment, 718 were free from diabetic retinopathy at baseline. Baseline retinal traits including vessel widths, tortuosity (curvature) and fractal dimensions (network complexity), were quantified using fundus camera images and semiautomated software, and analysed using logistic regression for their association with incident diabetic retinopathy over 10 years.

**Results:**

The incidence of diabetic retinopathy was 11.4% (*n* = 82) over 10 years. After adjustment for a range of vascular and diabetes-related risk factors, both increased venular tortuosity (OR 1.51; 95% CI 1.15, 1.98; *p* = 0.003) and decreased fractal dimension (OR 0.75; 95% CI 0.58, 0.96; *p* = 0.025) were associated with incident retinopathy. There was no evidence of an association with arterial tortuosity, and associations between measurements of vessel widths and retinopathy lost statistical significance after adjustment for diabetes-related factors and vascular disease. Adding venular tortuosity to a model including established risk factors for diabetic retinopathy (HbA_1c_, BP and kidney function) improved the discriminative ability (C statistic increased from 0.624 to 0.640, *p* = 0.013), but no such benefit was found with fractal dimension.

**Conclusions/interpretation:**

Increased retinal venular tortuosity and decreased fractal dimension are associated with incident diabetic retinopathy, independent of classical risk factors. There is some evidence that venular tortuosity may be a useful biomarker to improve the predictive ability of models based on established retinopathy risk factors, and its inclusion in further risk prediction modelling is warranted.

**Graphical abstract:**

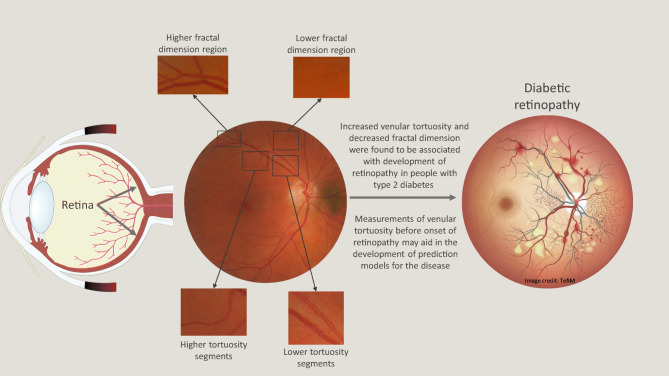

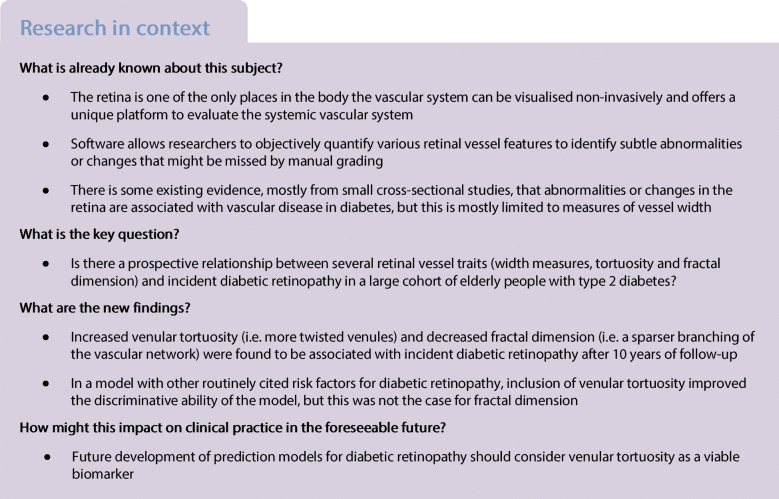



## Introduction

The microvasculature of the retina offers a unique opportunity for research of the systemic vascular system as it is one of the only locations in vivo where it is possible to visualise the vasculature of the human body non-invasively [1]. Diabetic retinopathy is one of the earliest microvascular complications of diabetes and is highly prevalent, with a conservative estimate in adults with diabetes of around 29%, and sight-threatening retinopathy affecting 4.4% [2]. It is well known that providing early treatment for diabetic retinopathy can improve visual outcomes [3], such that screening programmes are commonplace in many countries. However, these programmes can only detect prevalent disease, so there is a need for more sensitive screening for prepathological stages of retinopathy as well as improved stratification to help clinicians understand which patients are more likely to progress to sight-threatening stages. One area that is receiving increasing attention is morphological analysis of the appearance of retinal venules and arterioles [4–6].

Several cross-sectional studies have investigated associations between measurements of retinal vessel traits on fundus camera images and diabetic retinopathy in older adults, and these studies provided some evidence for an association between abnormally wider retinal venular calibre and diabetic retinopathy [7–9]. However, longitudinal evidence for other quantifiable retinal traits, such as vessel tortuosity and fractal dimension, reveal additional signs of vascular health and disease is limited. Critically, there is a need for prospective studies to evaluate if retinal vascular measurements could be used as biomarkers to predict subsequent development of diabetic retinopathy.

The objective of this analysis was to determine if retinal vessel traits measured at baseline in an ongoing, prospective study of older adults with type 2 diabetes were associated with incident diabetic retinopathy during 10-year follow-up, beyond other known risk factors for retinopathy.

## Methods

### Study design and participants

The Edinburgh Type 2 Diabetes Study (ET2DS) is a longitudinal cohort of older men and women based in Lothian, Scotland, designed to investigate the role of risk factors for vascular complications of type 2 diabetes. Methods have been previously reported in detail including in Price et al (2008) [10]. In brief, recruitment occurred in 2006–2007 when participants aged 60–75 years were randomly selected within sex and 5-year age bands from the Lothian Diabetes Register, with the final cohort shown to be largely representative of the target population of all older men and women with type 2 diabetes residing in Lothian [11]. All people within the Lothian Diabetes Register have a diabetes diagnosis according to WHO criteria, but in order to be enrolled in the ET2DS participants had to meet certain criteria to ensure a robust diagnosis of type 2 diabetes [12]. These criteria included that participants had to be taking oral glucose-lowering medications and/or insulin, or, if participants managed their diabetes through diet control methods alone, had to have an HbA_1c_ measure of >48 mmol/mol (6.5%) at the baseline clinic. Further investigation of clinical records was undertaken if there was concern of the diabetes status of a potential participant.

Ethical permission was granted by the Lothian Medical Research Ethics Committee at baseline and follow-up, and all participants gave written informed consent at recruitment and prior to subsequent clinic attendance. Data were collected at designated research clinics as well as through linkage to routine medical and death records, at baseline and during follow-up.

### Baseline data collection

Baseline fasting blood samples were used to measure total serum cholesterol, HDL-cholesterol and HbA_1c_, and early morning urine samples to measure creatinine and albumin. Height, weight, and systolic and diastolic brachial blood BPs were measured using established standard operating procedures by trained researchers, and self-administered questionnaires were used to collect data on diabetes history and treatment, smoking habits, medications and comorbidities. For cardiovascular and cerebrovascular events, answers from baseline questionnaires on medical history, the WHO chest pain and Edinburgh Claudication questionnaires, and results from a standard 12-lead ECG were combined with routine hospital discharge data [11] to define macrovascular disease (one or more of myocardial infarction, angina, stroke or transient ischaemic attack).

To determine retinopathy status at baseline and obtain retinal photographs to assess quantitative traits, all study participants were invited to an eye appointment within 3 weeks of their original baseline clinic appointment [13]. Mydriasis was achieved using 1% tropicamide drops and standard seven-field non-stereoscopic retinal colour photographs were taken of both eyes at 35° using a high-resolution TOPCON TRC-50FX digital retinal camera (Topcon Optical Company, Tokyo, Japan). The images were taken by a single, specially trained medical photographer. Retinopathy grading was undertaken by two trained optometrists, working independently, using all seven fields of both eyes and a predefined protocol using Early Treatment Diabetic Retinopathy Study (ETDRS) criteria [14]. An ETDRS grade of ≥20 in any field of either eye was considered as presence of diabetic retinopathy at baseline.

### Incident retinopathy

To determine incident retinopathy, results of retinopathy grading by the Scottish Diabetic Retinopathy Screening Programme (Scottish DRS) were requested, which operates an annual screening programme for all people in Scotland with diabetes. We were able to request all screening data through the 10-year follow-up period, which helped to reduce bias due to attrition. After pupil dilation using 1% tropicamide, images were taken of both eyes with a TOPCON NW8 fundus camera with Nikon D700 digital backs at a 45–50° angle. Photography and grading were performed by specially trained and accredited medical photographers using a grading scheme that ranges from R0 (no diabetic retinopathy) to R4 (proliferative diabetic retinopathy) [15]. Grade R1 is considered mild, R2 is observable, R3 is referable and R4 is proliferative. For this analysis, incident retinopathy was considered any grade R1 or above during the follow-up period. It is not uncommon for a person that has received a grade of R1 to then revert to R0 at another grading because of artefacts in the imaging, healing of microaneurysms without further progression of retinopathy or other reasons. Because of this, if a participant received a grade of R1 but no higher during the follow-up period, a grade of R1 at a minimum of two consecutive screenings was required for them to be considered to have incident retinopathy. A participant was also determined to have incident retinopathy if there were laser photocoagulation scars present (an indication of prior treatment for diabetic retinopathy).

### Retinal vessel trait measurements

Retinal vessel traits were measured at baseline by a single researcher (E. Sandoval Garcia), trained specially by an expert senior researcher (T. J. MacGillivray), using the Vascular Assessment and Measurement Platform for Images of the Retina (VAMPIRE) software (version 3.1.0, Universities of Edinburgh and Dundee, UK) [16]. The right eye image was analysed, unless unsuitable because of poor image quality leading to hazy or obscured views of the retinal vasculature, in which case the left eye image was used. Measures of inter- and intragrader agreement were made with involvement of a third, specially trained researcher (R. B. Forster). Measurements were taken using the optic disc-centred field.

To analyse an image, the user uploads it to the software upon which the boundary of the optic disc and the position of the fovea are detected automatically. The user can adjust these features if necessary (e.g. the optic disc in some patients may be unclear because of pathology or the fovea may have been masked by poor illumination at image acquisition). The software then automatically generates a map of the retinal vasculature and attempts to classify vessels as arterioles or venules, which can be edited by the user if some vessels are labelled incorrectly. A final measurement step is undertaken to generate quantitative traits.

The traits assessed for this analysis include vessel calibre, or width, measured as summary indices (central retinal arterial equivalent [CRAE] and central retinal venular equivalent [CRVE]), tortuosity, which describes the degree of curvature of a vessel (i.e. how much the vessels twist and turn, arterial and venular separately), and total fractal dimension, which describes how the vascular pattern fills a two-dimensional space and is thus a measure of complexity or sparsity of the vascular network. For CRAE and CRVE, the software identifies the six widest arterioles and venules that cross a standardised region called zone B [16]. Tortuosity is calculated using the six widest arteriole and venule vessels crossing another standardised region, zone C [17]. Fractal dimension was measured by multifractal analysis using the generalised sand box method, which generates an estimation of fractal dimension of several scales from zone C of a binary map [18, 19].

### Statistical analysis

All variables were checked for normality and the following were transformed prior to analysis: duration of diabetes, albumin/creatinine ratio (ACR), tortuosity and fractal dimension. All of these except fractal dimension were log transformed using the natural log. Fractal dimension, which was only slightly skewed, was modified using rank transformation. In addition, fractal dimension was standardised for use in regression modelling because it is a continuous, unitless measure that falls between 1 and 2, and can be difficult to interpret in an unstandardised form. Values were standardised using the mean and SD to produce a scaled variable with a mean of 0 and SD of 1 (values maintain the same relationship to one another as the unscaled variable). The resultant OR can be interpreted as the odds given an increase of one SD.

To evaluate reliability and agreement of the measured retinal traits, interclass correlation coefficients (ICC) were used to measure intra- and intergrader agreement. A two-way mixed effects design was used to evaluate the mean of two graders or two measurements for consistency [20]. For baseline measurements, 3% (*n* = 30) of participants were used for the analysis of inter- and intragrader reliability.

Variables of interest were initially evaluated using Welch’s unpaired two-sample *t* test for continuous variables, or Wilcoxon test if assumptions were not met, and Pearson’s χ^2^ test with Yate’s continuity correction for categorical variables.

Logistic regression analyses were used to evaluate the relationship between retinal vessel traits and incident retinopathy, and the model was built by adding blocks of covariates to better understand the impact of the variables within the model. First, an unadjusted model was generated, then age and sex were controlled for, followed by cardiometabolic risk factors (BMI, smoking status, systolic BP, and total cholesterol:HDL-cholesterol ratio), then the addition of diabetic risk factors (HbA_1c_, duration of diabetes and diabetic treatment type) and finally vascular disease risk factors were added (a composite history of macrovascular disease as well as ACR). When measuring vessel width, arterial and venular vessels were evaluated separately, as CRAE or CRVE, but the corresponding value for the opposite measure was added as a covariate in the final model because of the large amount of shared variance. If there was no evidence of an association at the unadjusted or age- and sex-adjusted stages, the analysis was halted to avoid the risk of findings through multiple testing. Only cases with no missing data were included in logistic regression, and we planned to investigate any covariates with missing data exceeding 3.5% of total cases.

Subgroup analysis was planned to evaluate the different severities of retinopathy gradings. Grading groups would include mild retinopathy (R1), moderate (R2 and R3) and proliferative retinopathy (R4). However, the very small number of incident retinopathy cases between R2 and R4 meant that such analysis was not undertaken. Ethnicity subgroups were not evaluated as the ET2DS consists of 98% white British participants, reflecting the general population at baseline.

Initially, a Cox proportional hazards regression model was considered, but the proportional hazards assumption was violated and could not be resolved, so logistic regression was the preferred approach.

If any vessel traits were found to be independently associated with incident retinopathy they would be evaluated, in combination with the most highly predictive risk factors (HbA_1c_, systolic BP and ACR) [2, 21–24], using Harrell’s concordance statistic or C statistic to see if the model was improved, which is used to evaluate the discriminative ability of the model. The models would also be evaluated using the likelihood ratio test and Akaike’s information criterion (AIC).

A small subset of the total ET2DS population was evaluated for changes in retinal vessel traits, measuring retinal traits at baseline and then at the latest follow-up image available, suitable for analysis, using retinal images from the Scottish DRS. To test for a difference in the retinal trait from baseline to follow-up, Welch’s two-sample paired *t* test for continuous variables was used, or a Wilcoxon test if assumptions were not met.

All final models were evaluated for concerns with multicollinearity and linearity. Goodness of fit was evaluated using the Hosmer–Lemeshow test, residuals were inspected for outliers and influential cases, and interaction with predictor variables was investigated.

A two-sided *p* value ≤0.05 was used to indicate evidence of statistical significance. Logistic regression results were presented as OR and 95% CI, and all statistical analyses were performed using R version 3.5.1 [25].

## Results

**Fig. 1:**
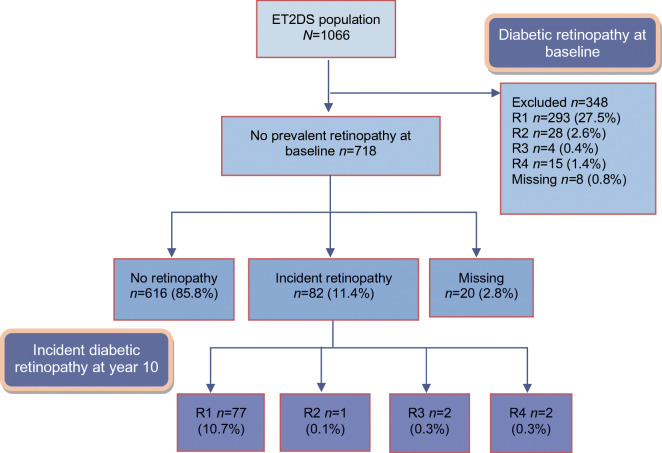
Diabetic retinopathy in the ET2DS population at baseline and year 10

At baseline, 340 of 1066 participants had prevalent diabetic retinopathy and eight did not have sufficient data to determine their retinopathy status (they did not attend the baseline ET2DS eye screening and did not attend a Scottish DRS screening around the time of ET2DS baseline). During 10 years of follow-up, 82 participants (11.4%) of 718 with no baseline prevalent disease developed retinopathy (Fig. 1). The median number of screening visits was 8 with a range of 1 to 12. The vast majority had mild disease with 77 graded R1, one R2 and two R3 as well as two participants who had R4 graded disease.

Overall, participants with incident retinopathy were similar to those without for the key baseline variables reported in Table 1. However, those that developed retinopathy had increased HbA_1c_ (60 mmol/mol vs 55 mmol/mol (7.6% vs 7.2%)). Both vessel width measures, CRAE and CRVE, and fractal dimension were reduced in those with incident retinopathy, while venular tortuosity was increased.

**Table 1:** Baseline characteristics and retinal vessel traits by incident retinopathy status

Characteristic	*n*	Incident retinopathy (*n*=82)	No incident retinopathy (*n*=616)	*p* value
Age, years	718	67.4 (4.1)	67.9 (4.2)	0.222
Sex, men	718	40 (48.2%)	300 (48.7%)	0.920
Ever smoker, yes	718	40 (48.2%)	335 (54.4%)	0.337
BMI, kg/m^2^	718	31.0 (5.9)	31.5 (5.6)	0.720
Systolic BP, mmHg	716	134.7 (14.8)	132.9 (15.9)	0.286
Total cholesterol:HDL-cholesterol ratio	714	3.6 (1.2)	3.5 (1.1)	0.564
HbA_1c_	712			0.006
mmol/mol		60 (10.9)	55 (10.9)	
%		7.6 (1.0)	7.2 (1.0)	
Duration of diabetes, years, median (IQR)	712	5.0 (3.0–9.0)	5.0 (3.0–9.0)	0.515
Diabetes treatment	717			0.186
Diet controlled (%)		13 (15.7%)	152 (24.7%)	
Tablets (%)		60 (72.3%)	399 (64.8%)	
Insulin (%)		10 (12.0%)	63 (10.2%)	
Macrovascular events, yes	718	25 (30.1%)	206 (33.4%)	0.625
ACR, mg/mmol, median (IQR)	713	1.2 (0.9–2.1)	1.1 (0.7–1.7)	0.054
CRAE, pixels	694	32.03 (3.71)	33.03 (3.78)	0.018
CRVE, pixels	694	43.54 (4.36)	44.53 (5.04)	0.044
Arterial tortuosity	694	−10.11 (1.26)	−10.11 (1.11)	0.914
Venular tortuosity	694	−9.71 (1.06)	−10.01 (0.87)	0.016
Fractal dimension	686	1.74 (0.08)	1.75 (0.07)	0.048

Values are mean (SD) or *n* (%) unless otherwise stated

Repeatability for retinal traits between graders was good, with ICC values between 0.81 (95% CI 0.61, 0.91) for fractal dimension to 0.91 (95% CI 0.81, 0.96) for arterial tortuosity. Intra-grader ICC values were also very good, ranging from 0.95 (95% CI 0.90, 0.98) for CRVE to 0.98 (95% CI 0.96, 0.99) for CRAE.

Association between retinal vessel traits and diabetic retinopathy.

Results of logistic regression assessing the association between the retinal vessel traits and diabetic retinopathy are shown in Table 2.

**Table 2:** Association between retinal vessel traits and incident retinopathy: logistic regression

	CRAE		CRVE		Arterial tortuosity		Venular tortuosity		Fractal dimension	
Model	OR (95% CI)	*p* value	OR (95% CI)	*p* value	OR (95% CI)	*p* value	OR (95% CI)	*p* value	OR (95% CI)	*p* value
1^a^	0.93 (0.87, 0.99)	0.028	0.95 (0.91, 1.00)	0.058	0.99 (0.81, 1.22)	0.946	1.43 (1.11, 1.84)	0.005	0.80 (0.63, 1.01)	0.059
2^b^	0.93 (0.87, 0.99)	0.023	0.95 (0.91, 1.00)	0.047	0.99 (0.80, 1.22)	0.923	1.44 (1.12, 1.86)	0.004	0.79 (0.62, 1.00)	0.050
3^c^	0.93 (0.87, 0.99)	0.030	0.95 (0.91, 1.00)	0.048			1.49 (1.16, 1.93)	0.002	0.80 (0.63, 1.02)	0.073
4^d^	0.93 (0.87, 1.00)	0.038	0.95 (0.91, 1.00)	0.051			1.57 (1.21, 2.04)	0.001	0.76 (0.60, 0.98)	0.033
5^e^	0.95 (0.87, 1.03)	0.212	1.00 (0.58, 1.75)	0.999			1.51 (1.15, 1.98)	0.003	0.75 (0.58, 0.96)	0.025

^a^Model 1: unadjusted

^b^Model 2: age- and sex-adjusted

^c^Model 3: Model 2 + cardiometabolic risk factors (BMI, smoking status, systolic BP and total cholesterol:HDL-cholesterol ratio)

^d^Model 4: Model 3 + diabetes-related risk factors (HbA_1c_, duration of diabetes and diabetic treatment type)

^e^Model 5: Model 4 + vascular disease (composite CVD and ACR) + CRAE or CRVE when analysing CRAE or CRVE; CRAE and CRVE *n*=655 (*n*=63 removed because of missing data), tortuosity *n*=659 (*n*=59 observations removed because of missing data) and fractal dimension *n*=641 (*n*=77 observations removed because of missing data)

### CRAE

In the unadjusted model, decreased CRAE (i.e. narrower arterioles) was associated with incident retinopathy (OR 0.93; 95% CI 0.87, 0.99; *p* = 0.028). This relationship was maintained after multivariable adjustment for age and sex, as well as after adding cardiometabolic and diabetes-related risk factors, with little change in the point estimate at each step. However, the relationship lost statistical significance after vascular disease history and CRVE were incorporated (OR 0.95; 95% CI 0.87, 1.03; *p* = 0.212).

### CRVE

In unadjusted and multivariable-adjusted models, evidence for an association between decreased CRVE (i.e. narrower venules) and incident retinopathy was weak (unadjusted OR 0.95; 95% CI 0.91, 1.00; *p* = 0.058; OR after adjustment for age, sex, cardiometabolic risk factors 0.95; 95% CI 0.91, 1.00; *p* = 0.048). The association was not evident after further adjustment for diabetes-related risk factors, vascular risk factors and arterial width.

### Arterial and venular tortuosity

Arterial tortuosity was not associated with incident retinopathy (unadjusted OR 0.99; 95% CI 0.81, 1.22; *p* = 0.946). There was evidence of a relatively strong association between increased venular tortuosity (i.e. more twisted venules) and incident retinopathy, OR 1.43 (95% CI 1.11, 1.84; *p* = 0.005) and this was maintained after each block of covariates was added (full multivariable-adjusted OR 1.51; 95% CI 1.15, 1.98; *p* = 0.003).

### Fractal dimension

Despite little evidence of an association between decreased fractal dimension (i.e. a sparser vascular network) and incident retinopathy in initial models (unadjusted OR 0.80; 95% CI 0.63, 1.01; *p* = 0.059; age and sex-adjusted OR 0.79; 95% CI 0.62, 1.00; *p* = 0.050), evidence of an association was evident after adjustment for cardiometabolic and diabetes risk factors (OR 0.76; CI 0.60, 0.98; *p* = 0.033) and in full multivariable analysis (OR 0.75; 95% CI 0.58, 0.96; *p* = 0.017).

### Prediction modelling for venular tortuosity and fractal dimension

Venular tortuosity and fractal dimension were added, separately, to a model that contained the most highly cited risk factors for diabetic retinopathy (HbA_1c_, ACR and systolic BP). For venular tortuosity the discriminative ability of the resultant model improved from 0.624 to 0.640, based on the C statistic, and was confirmed by the Likelihood ratio test, *p* = 0.013 and AIC measure, which decreased from 487.65 to 483.43, showing a statistically significant improvement in the model. However, when fractal dimension was added, the C statistic decreased from 0.625 to 0.621 (*p* value 0.048 for likelihood ratio test), indicating the addition did not improve the model. Values are shown in Table 3.

**Table 3:** Discriminative changes in model with addition of tortuosity and fractal dimension

Change	Venular tortuosity			Fractal dimension		
	C statistic	AIC	Likelihood ratio test *p* value	C statistic	AIC	Likelihood ratio test *p* value
Base model	0.624	487.65	0.013	0.625	485.22	0.048
Base model + retinal trait	0.640	483.43		0.621	483.32	

Base model is made up of HbA_1c_, systolic BP and ACR

Model fit was evaluated by comparing the C statistic and AIC between the unadjusted model and the fully adjusted model and the Hosmer–Lemeshow goodness of fit test was used to look for evidence of poor model fit. All models demonstrated good fit, and when evaluating residuals there was no evidence of strong outliers or influential cases, and no indication of interaction.

### Change in retinal traits over time

In a subset of the cohort, *n* = 170, we evaluated if there was evidence of change in the individual retinal traits over time using two timepoints: baseline and closest to the 10-year follow-up time point. We identified no differences in width or tortuosity, but a statistically significant decrease in fractal dimension. This subset was not powered to evaluate those with incident diabetic retinopathy, but of the 19 cases that were included in this subset, they followed the same pattern: a decrease in fractal dimension but no difference in widths or tortuosity.

## Discussion

Increased venular tortuosity and decreased fractal dimension were independently associated with incident diabetic retinopathy above and beyond other known risk factors. There was also an association between narrower vessel widths with diabetic retinopathy, but the associations were no longer evident when all covariates were added to the model. In exploratory analyses, venular tortuosity was shown to improve upon the discriminative ability of a model that included the most cited risk factors for retinopathy, suggesting a promising biomarker for future in-depth prediction modelling.

There are several strengths in our analysis. The ET2DS is a well-established, representative cohort with a large amount of information on participants at baseline, which increases the generalisability of the findings, enabling multivariable adjustment and long-term follow-up for incident retinopathy. During data collection, systematic and random error were reduced through the use of standard operating procedures, as well as linkage to routine data from high-quality sources, including diabetic retinopathy screening [26, 27]. Semiautomatic retinal vessel assessment using VAMPIRE software allowed for reduction in random error and produced reliable measurements efficiently.

There were also some unavoidable limitations and findings that must be treated with caution. In the model used to test the addition of venular tortuosity and fractal dimension for improvement in discrimination, the C statistic did not meet the general convention of a ‘good model’ (cut-off 0.70) and the ET2DS was not large enough to appropriately develop a full prediction model. Notably, amongst previous studies considering the development of a prediction tool for diabetic retinopathy, none have been reliably replicated and implemented in clinical practice [28]. Our findings suggest that future work in this area should consider inclusion of ‘novel’ retinal traits, especially venular tortuosity.

Our study was further limited by insufficient power to allow subgroup analysis by retinopathy severity grading (only two cases of incident R3- and two cases of incident R4-graded retinopathy). For replication of these findings, it would be ideal to conduct this analysis in a cohort with newly diagnosed diabetes, in order to capture more cases of incident severe retinopathy during follow-up, as there is evidence that those that develop clinically meaningful diabetic retinopathy are more likely to have a retinopathy diagnosis earlier [29].

As the number of studies reporting on retinal vessel traits increases, there is increasing concern about direct comparison between different measurement platforms being used because of heterogeneity in algorithms and methods. A recent study carried out a direct comparison of a wide number of vessel measurements between VAMPIRE and Singapore ‘I’ Vessel Assessment (SIVA; National University of Singapore, Singapore) and found poor agreement between the software platforms [19]. Another study, which compared results for CRAE and CRVE between SIVA, Interactive Vessel Analysis (University of Wisconsin–Madison, WI) and retinal analysis (Department of Ophthalmology and Visual Science, University of Wisconsin–Madison), also found poor agreement, but associations with systemic factors including age, BP and cholesterol were similar between applications [30]. Yip et al suggested that use of a conversion algorithm between the platforms could help overcome differences.

There is a current debate around retinal vessel widths changing during different phases of the cardiac cycle that should be considered when interpreting the results of this type of study [31]. However, a study evaluating observer bias did not find a difference when using ECG-synced vs non-synced images [32]. Also, it is debatable whether the software packages are sensitive enough to pick up such slight changes in width as the resolution of a fundus camera is roughly 7 μm and pulsatile changes are likely to be less than 1 μm in size [1].

There have been similar studies to ours that have evaluated the relationship between venular tortuosity and diabetic retinopathy. In one longitudinal analysis of the Wisconsin Epidemiological Study of Diabetic Retinopathy (WESDR) with 1370 people with type 2 diabetes, increased venular tortuosity was associated with incident proliferative diabetic retinopathy, but not any incident retinopathy [33]. Several other studies did not find any statistically significant associations but interestingly, for all these studies, their direction of effect was consistent with the findings of the ET2DS analysis [6, 34, 35]. It should be noted that these studies were either in younger type 1 diabetes populations or had mixed aetiology of diabetes, and in some cases analysis methods were quite different.

Two observational studies, in younger people with type 1 diabetes, also found an association with lower fractal dimension and proliferative diabetic retinopathy; one study was cross-sectional [5] and the other longitudinal [4]. There have been other similar longitudinal studies that found no evidence of an association [33], and one study found an association between higher arteriolar fractal dimension and diabetic retinopathy [35]. Using different analysis and computational methods, two recent studies showed the ability of fractal dimension to assist in identification of very early retinal disease [36, 37]. Both studies were conducted in people with retinopathy, so prediction was not the aim. However, these studies provide evidence that could help determine how the retinal vasculature is changing in the very early stages of disease.

Two recent studies have provided evidence on using retinal vessel traits in prediction modelling for retinopathy. Both used principle components analysis to combine multiple measures and extract a single component. One study found an improvement in the discriminative ability of modelling for diabetic retinopathy [35], agreeing with the findings from this analysis, while the other analysis found no change [33]. This also touches on the progression in the field of using reductive statistical methods to combine information from multiple retinal traits, which should be considered in future analyses and has been done for other disease outcomes.

Measurement in change in the retinal traits over time is a novel approach and the data presented in this paper represent the longest follow-up, mean of 9 years, in people with type 2 diabetes, although our study was not well powered to evaluate the association with diabetic retinopathy. One other study, from the WESDR population, evaluated changes over 6 years and found a narrowing of arterioles and widening of venules, but reported no changes in fractal dimension [9].

There is currently no exact mechanistic explanation for the changes seen in retinal vessels prior to pathology and they would most likely differ for different vessel traits, but haemodynamic changes, especially in more thinly walled venular vessels, are probably key. Previous studies have shown that changes in brachial BP from exercise can lead to incremental changes in retinal pressure as a normal process of autoregulation, but these processes may break down in older people and those with diabetes [38, 39]. It is therefore possible that with sustained blood flow changes not only from chronically increased BP, but also age and damage caused by hyperglycaemia, the smaller vessels in the retina undergo cumulative alterations. There is also a possible genetic underpinning as recent data from the Genetics of Diabetes Audit and Research Tayside and Scotland (GoDARTS) study has shown a genetic link with differences in venular tortuosity that is also associated with risk factors for coronary artery disease [40].

Fractal dimension in relation to diabetic retinopathy is complex, because throughout the course of the disease there are cumulative vascular changes that may create opposing findings in fractal dimension. Fractal dimension may change dramatically from moderate non-proliferative disease to proliferative retinopathy, characterised by new vessel growth, and may be part of the reason some studies find increased fractal dimension to be associated with diabetic retinopathy [41, 42]. Unfortunately, in this analysis there were not enough people with proliferative retinopathy to undertake robust analysis.

In conclusion, there is gathering evidence from this analysis and other similar studies that retinal vessel traits have the potential to assist in the prediction of vascular outcomes such as diabetic retinopathy. Not only could earlier identification of retinopathy help individuals maintain healthy vision for longer, but there is also currently a debate around optimal frequency of retinopathy screening. Findings such as ours could assist screening programmes in determining who is most at risk of developing sight-threatening retinopathy in order to inform stratified screening intervals [26]. Such adjustments could help reduce burden on the healthcare services and prioritise people with the most need. Although our findings are suggestive of a possible benefit from incorporating venular tortuosity measures into a risk prediction tool, further research is needed to create a robust prediction tool that can be used effectively in clinical practice. In addition to validating the results of this research, future research should focus on overcoming the heterogeneity between findings from different software types, evaluating change in the retinal traits over time as well as developing cut points within venular tortuosity to help clinicians indicate increased risk of diabetic retinopathy.

## Data Availability

Because of the sensitive nature of the clinical data the full dataset is not publicly available, but the protocol and statistical analysis plan are available by request to the corresponding author.

## Abbreviations

ACR: Albumin/creatinine ratio
AIC: Akaike’s information criterion
CRAE: Central retinal arteriole equivalent
CRVE: Central retinal venular equivalent
ET2DS: Edinburgh Type 2 Diabetes Study
ICC: Interclass correlation coefficient
Scottish DRS: Scottish Diabetic Retinopathy Screening Programme
SIVA: Singapore ‘I’ Vessel Assessment
VAMPIRE: Vascular Assessment and Measurement Platform for Images of the Retina
WESDR: Wisconsin Epidemiological Study of Diabetic Retinopathy
